# Intersection of Autonomic Dysfunction and Parkinson’s Disease: Insights Into Neurogenic and Classical Orthostatic Hypotension

**DOI:** 10.7759/cureus.88487

**Published:** 2025-07-22

**Authors:** Jamir Pitton Rissardo, Masoumeh Rashidi, Fatemeh Rashidi, Khalil I Hmedat, Ibrahim Khalil, Hania Moharam, Mallak Bahar, Ali Dway, Omesh Prathiraja, Ana Leticia Fornari Caprara, Maleesha Jayasinghe

**Affiliations:** 1 Neurology, Cooper University Hospital, Camden, USA; 2 Medical School, Nanjing Medical University, Nanjing, CHN; 3 Internal Medicine, Faculty of Medicine, Alexandria University, Alexandria 5372066, Egypt, Hebron, PSE; 4 Neurological Surgery, Alexandria faculty of medicine, Alexandria, EGY; 5 Medicine and Surgery, Nanjing medical university, Nanjing, CHN; 6 Medical Education, Nanjing medical university, Casablanca , MAR; 7 Medical School, Faculty of Medicine, Al-Andalus University, Tartus, SYR; 8 Medicine and Surgery, Nanjing Medical University, Nanjing, CHN; 9 Neurology, Universidade Federal de Santa Maria, Santa Maria, BRA; 10 Internal Medicine, Nanjing Medical University, Nanjing, CHN

**Keywords:** autonomic failure, baroreflex dysfunction, dysautonomia, neurogenic orthostatic hypotension, nocturnal hypertension, non-motor symptom, parkinson’s disease, postprandial hypotension, prodromal, supine hypertension

## Abstract

Neurogenic orthostatic hypotension (nOH) and classical orthostatic hypotension (OH) are prevalent non-motor manifestations of Parkinson’s disease (PD). They can significantly impact quality of life, increasing the risk of falls, cognitive decline, and functional impairment. Despite the high prevalence and clinical impact of neurogenic orthostatic hypotension and OH in PD, no comprehensive consensus integrates recent advances in pathophysiology, diagnostic tools, and personalized treatment. This review synthesizes current evidence to bridge this gap, offering a practical framework for clinicians to improve patient outcomes. Neurogenic orthostatic hypotension in PD results from complex interactions between central and peripheral autonomic dysfunction, alpha-synuclein accumulation, baroreflex failure, and medication effects. Its prevalence increases with disease progression and age. Clinical evaluation remains the cornerstone of diagnosis, supported by specialized testing such as the active standing test, ambulatory blood pressure monitoring, and autonomic function assessments. Management requires a tailored approach, combining non-pharmacologic strategies, such as fluid and salt intake optimization, compression garments, and physical counter-maneuvers, with pharmacological treatments, including midodrine, droxidopa, and fludrocortisone. Emerging therapies and ongoing clinical trials offer promising avenues for future interventions. Early recognition and individualized management of OH are critical in PD care.

## Introduction and background

Parkinson’s disease (PD) is a progressive, multi-system neurodegenerative disease affecting people, mainly in the later years of life. The nonmotor symptoms of PD can be categorized as cognitive and psychiatric disturbances, sleep disturbances, sensory symptoms, and autonomic dysfunction. Autonomic dysfunction, which has emerged as an important aspect of nonmotor dysfunction and is present in both the early and later stages of PD, covers a broad spectrum that includes gastrointestinal, urological, sexual, thermoregulatory, and cardiovascular [1].

Cognitive and psychiatric disturbances represent some of the most prevalent non-motor manifestations of PD, potentially emerging at any disease stage, including in prodromal phases before motor symptoms appear. These manifestations, which include depression, anxiety, apathy, and psychosis, frequently overlap and interact with each other. Notably, depression often coexists with anxiety disorders, while apathy may present either independently or as part of depressive syndromes. The complex interplay between these symptoms and autonomic dysfunction in PD suggests shared underlying mechanisms, including central autonomic network involvement and neurodegenerative processes affecting both cognitive and autonomic pathways. Clinically significant depression and anxiety affect approximately 30-35% of PD patients [2]. Psychosis is reported in 25-40% of cases, with visual hallucinations occurring in 15-30%, non-visual hallucinations (including auditory, tactile, and olfactory) in up to 35%, and delusions in about 4% of patients [2]. The effect of medications should also be mentioned. Cognitive deterioration and dementia are common in PD and can manifest at any stage of the disease, either early or late in its course. Early cognitive changes often include deficits in executive function, visuospatial dysfunction, impaired speech fluency, and memory impairment, which may be subtle but progressively impact daily functioning [3].

In addition to psychiatric disturbances, sleep abnormalities are another major nonmotor burden in PD. The neuropathology of PD is known to affect anatomical structures and central neurotransmitters involved in modulating the physiological sleep cycle. Patients with PD also tend to have more shallow sleep and frequent awakenings in the night due to other symptoms, such as difficulties turning around in bed, frequent nocturia, and nocturnal tremors. Depression may also lead to fragmented sleep [3,4].

Apart from the nonmotor symptoms, autonomic dysfunction can present before the diagnosis becomes apparent with disease progression or be induced by medications. It is believed that the involvement of both the central and peripheral postganglionic autonomic nervous systems is the cause of these symptoms [5]. Gastrointestinal symptoms are prevalent, such as constipation, which is conceded to be the most common complaint. Sialorrhea occurs in over 50% of early PD patients [6], and bedside diurnal drooling causes embarrassment and increases the risk of aspiration pneumonia, postprandial fullness, and gastric retention. Difficulty in rectal evacuation because of rectal sphincter dysfunction can also be observed [7].

Along with gastroparesis and dysphagia, which occur in 11-81% of PD patients [8]. Almost half of PD patients show weight loss during disease progression, which may be related to these gastrointestinal symptoms or medications such as levodopa [9]. Urinary symptoms and erectile dysfunction are common in males, with control disturbances including urinary frequency, urgency, and incontinence [10,5]. Frequent nocturia is reported by 60% of patients due to detrusor overactivity, which results in a significant impact on the individual's quality of life, so most patients should undergo a full urodynamic investigation, including cytometry, flowmetry, and ultrasonography, before treatment is initiated [11].

Among the various components of autonomic dysfunction, cardiovascular disturbances are particularly impactful due to their potential for acute complications. Cardiovascular autonomic dysfunction in Parkinson’s disease (PD) can involve both pre-ganglionic and post-ganglionic lesions, and the interplay between these contributes to the development of classical orthostatic hypotension (OH) and neurogenic orthostatic hypotension (nOH). OH is defined as a reduction in systolic blood pressure (SBP) of at least 20 mmHg or diastolic blood pressure (DBP) of at least 10 mmHg within three minutes of standing, as per consensus criteria (e.g., American Academy of Neurology (AAN) guidelines). As one of the autonomic disorders, OH can be thought of as being either primary or secondary, which may occur in association with any condition of autonomic neurocirculatory dysfunction that causes a significant decline in blood or extracellular fluid volume, making a person bedridden or immobile for an extended period [12]. When performing the tilt table test, some centers consider a systolic drop of ≥30 mmHg as significant, while others use a ≥15 mmHg cutoff; the test aims to eliminate the leg muscle pump effect, which is usually active during standing [13]. In PD, nOH is typically present, with inadequate neurocirculatory responses to postural changes such as baroreflex failure and impaired release of norepinephrine [14]. At the same time, post-prandial hypotension (PPH) and exercise-induced hypotension can also occur in patients with PD. Postprandial postural hypotension (PPPH) occurs after consuming large, carbohydrate-heavy meals due to mesenteric vasodilation and impaired sympathetic compensation in PD, and may develop within 15 minutes after eating while persisting for up to three hours [15]. Elderly PD patients may be most susceptible to this phenomenon, which can be avoided by reducing carbohydrate intake and eating smaller, more frequent meals [15].

On the other hand, supine hypertension occurs in approximately 50% of individuals with neurogenic orthostatic hypotension. It is defined as a SBP ≥ 140 mmHg and DBP ≥ 90 mmHg after at least five minutes of rest in the supine position [16]. The decision of whether to treat supine hypertension or not in an individual with PD is challenging, as the consequences of nOH, such as falling and syncope, are more immediate and acutely dangerous than the delayed potential complications of supine hypertension. Non-dipping is the loss of any decrease in nocturnal BP. Although limited, studies on non-dipping in PD report that up to 88% of patients exhibit a non-dipping blood pressure pattern [17]. Also, it was found to be more prevalent in PD patients with OH than in those without.

This review offers a comprehensive analysis of the relationship between OH and neurogenic orthostatic hypotension in patients with PD, focusing on the epidemiology, diagnostic criteria, and clinical manifestations. It further explores the underlying pathophysiological mechanisms and critically evaluates current and emerging management strategies. Emphasizing the clinical relevance, the review highlights the impact of OH on the quality of life of patients living with PD.

## Review

Epidemiology

Prevalence and Incidence of Orthostatic Hypotension ​​​​​​

Although the prevalence of nOH in PD is relatively high, not all patients have symptoms of organ hypoperfusion, and only a third of patients (approximately 16%) have symptomatic nOH [18]. Between 5.7% and 64.9% of patients with PD have OH, and the prevalence of OH in PD increases with age and disease duration (Table 1).

**Table 1:** Epidemiological studies on the incidence and prevalence of orthostatic hypotension in Parkinson's disease BP: blood pressure; OH: orthostatic hypotension; PD Parkinson’s disease; PPMI: Parkinson’s progression markers initiative; nOH: neurogenic orthostatic hypotension; RR: relative risk; IV: intravenous; MIBG: meta-iodobenzylguanidine

References	Prevalence of OH in PD (%)^a^	Population Size	Notes
Senard et al. (1997) [19]	58.2	65	OH was asymptomatic in 38.5% and symptomatic in 19.8% of patients. Symptomatic OH correlated with disease duration, severity, and higher doses of levodopa and bromocriptine.
Allcock et al. (2006) [20]	49.7	175	OH was associated with cognitive decline and increased risk of falls.
Goldstein et al. (2008) [21]	64.9	77	Cardiac 6-[18F] fluorodopamine-derived radioactivity can be used to diagnose OH.
Haensch et al. (2009) [22]	48.3	58	MIBG scintigraphy is a sensitive tool for detecting early cardiac sympathetic denervation in PD, independent of OH and baroreflex failure.
Jamnadas-Khoda et al. (2009) [23]	42.0	50	OH frequently appears after tilting rather than standing and is often delayed (after 3 minutes)
Matinolli et al. (2009) [24]	52.5	120	Patients with OH showed greater postural sway while standing compared to those without OH, though mobility and walking speed remained unaffected.
Schmidt et al. (2009) [25]	22.0	32	During the Valsalva maneuver, the absence of the expected BP rise in phase IV, more frequent than in phase II, was common in PD, though its correlation with OH during tilt-table testing was only moderate.
Shibata et al. (2009) [26]	18.1	72	The coefficient of variation of RR intervals, an index of cardiac parasympathetic activity, predicts OH risk.
Ha et al. (2011) [27]	18.0	1125	Symptomatic OH was associated with older age, advanced disease stage, and longer disease duration.
Palma et al. (2015) [18]	50.0	210	Despite common OH in PD, symptoms vary; standing BP <75 mmHg guides treatment decisions against supine hypertension risk.
Merola et al. (2016) [28]	30.6	121	Among OH patients, 62.2% were symptomatic, and 37.8% were asymptomatic. Both groups had similar impairments in activities of daily living, worse than patients without OH.
Klanbut et al. (2018) [29]	22.0	100	Symptomatic OH was associated with older age and a history of hypertension. It was observed in 18% of patients, while asymptomatic OH was present in 4%.
Beah et al. (2024) [30]	5.7	907	In the PPMI early PD cohort, nOH was more prevalent than non-nOH, with a slight increase over time.

In individual studies that defined nOH by BP reduction criteria, the prevalence of nOH in patients with PD ranges from 10 to 65% [18,31]. A meta-analysis of 25 studies identified an estimated point prevalence of 30% [32]. The prevalence estimates of symptomatic nOH hypotension in patients with PD are also varied, with reported rates ranging from 16 to 89% in individual investigations [18,19,27,31]. A total of 907 PD patients with baseline orthostatic vitals were included in the most recent study. Using the Heart rate (HR)/SBP ratio to determine the prevalence of nOH and non-neurogenic OH in the Parkinson’s Progression Markers Initiative (PPMI), although the prevalence of nOH and non-neurogenic OH increased yearly (P = 0.012, chi-square), the increase was modest (baseline: 5.6% (95% CI: 4.3-7.3%); month 48: 8.6% [6.4-11.5%]). In comparison to PD patients without OH, nOH patients were older, and nOH was associated with more significant impairment of motor and independent functioning than the non-neurogenic OH/OH categories. Cognitive function and typical OH symptoms were generally worse in PD+OH [30].

In another study, OH, whether spontaneous (SOH) or levodopa-induced (LOH), was associated with older age, motor fluctuations, probable REM sleep behavior disorder, and greater autonomic, cardiovascular, and digestive dysfunction compared to non-OH patients. Both SOH and LOH were linked to increased disease severity and poorer quality of life, supporting the hypothesis that OH may serve as a clinical marker for the body-first subtype of PD [33]. Interestingly, the Hoehn and Yahr stage (OR 4.950) emerged as a strong predictor of OH risk, while the PDQ-39 score reflected a significant decline in quality of life, particularly in motor function, cognition, physical comfort, and daily living. The Hoehn and Yahr stage alone predicted OH with moderate accuracy (AUC = 0.679); however, predictive accuracy improved when combined with levodopa dosage and MDS-UPDRS Part III scores [34].

Other original studies, as summarized in Table 1, have investigated the prevalence of OH in PD. A cross-sectional study by Adhiyaman et al. found a prevalence of OH of 46.2% in 13 patients [35]. Another cross-sectional study by Allcock et al. reported a prevalence of 49.7% in 175 patients, and a cross-sectional study by Matinolli et al. found a prevalence of 52.5% in 120 patients [20,24]. Besides, Haensch et al. found a prevalence of 48.3% in 58 patients, while Jamnadas-Khoda et al. reported a prevalence of 22% in 50 patients [22,23]. Goldstein et al. found a prevalence of 64.9% in 77 patients, while Shibata et al. reported a prevalence of 18.1% in 72 patients [21,26]. Finally, Schmidt et al. found a prevalence of 38% in 26 patients [25]. It is important to note that the definitions of OH used in these studies varied, and the sample sizes were relatively small, making it difficult to draw firm conclusions about the prevalence of OH in PD.

Diagnosis

Clinical Manifestations

OH is commonly referred to as a sudden decrease in BP upon standing and is associated with different neurological symptoms in patients suffering from PD [13,36]. The pathophysiology of the neurological symptoms presented due to OH in PD primarily pertains to a reduction in blood flow toward the brain, occurring as an aftereffect of the inability of the body to maintain BP due to postural changes in the patient [13,37]. One of the most common symptoms is lightheadedness, often described as dizziness accompanied by a sensation of unsteadiness and feeling faint or about to lose consciousness [38,39]. Lightheadedness may severely impair an individual's ability to maintain an upright position while standing or walking for extended periods, making everyday activities particularly challenging [38,40,41].

Another common clinical feature of OH in PD is visual disruption [19,38]. These are often manifested as blurred or dimmed vision, whereby individuals cannot clearly distinguish between objects or the environment around them and find everyday tasks that require sharp visual acuity challenging [38,42]. This blurred vision tends to come suddenly when one stands and generally clears up when resuming a seated or lying position, immediately linking it to postural changes [43]. Diminished blood flow to the visual cortex in the brain is thought to be a primary contributor to these visual symptoms [44,45].

Many subjects with OH in PD complain of a sensation of cognitive slowing or “brain fog” when standing, which might conceivably engender significant functional impairment [41,46]. Mental clouding includes concentration problems, an inability to remember things, and generally slowed thought processes that eliminate spontaneity from daily life [46,47]. Concentrating on conversations and making decisions can become especially difficult, leading to frustration and inadequacy [46,48]. Notably, these cognitive deficits are often unnoticed by the affected individual but can be readily apparent to others [38,48,49].

In a study analyzing data from 453 PD patients in the Cincinnati Cohort Biomarkers Program, OH was not independently linked to excessive daytime sleepiness (EDS), as measured by the Epworth Sleepiness Scale. However, in cognitively impaired patients, OH was associated with depression, which in turn was strongly linked to EDS, suggesting an indirect pathway [50]. In another cross-sectional study of 82 patients with PD, nOH was independently associated with impaired gait and balance, including reduced gait speed, shorter stride length, longer postural transition times, and increased postural sway. These effects were more pronounced in patients with hemodynamically relevant nOH, with higher postural sway linked to a 7.9-fold increased fall risk [51].

This array of neurological symptoms, combined with OH in PD, testifies to the extent of inadequate blood supply to the brain, manifested by the impairment across various cognitive domains [45,52]. Although symptoms like dizziness and visual disturbances may be temporary and improve when lying down, they can significantly impact quality of life and increase fall risk [53]. Early detection of OH and timely management are crucial for reducing symptoms, enhancing quality of life, and promoting independence in people with PD [36,54].

Clinical Evaluation

**Figure 1:**
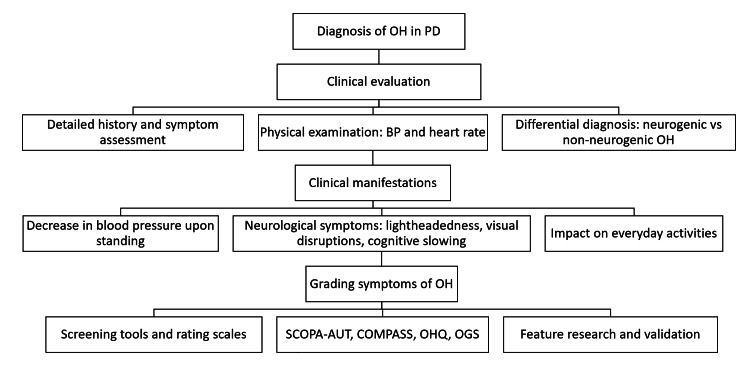
Diagnosis of orthostatic hypotension in Parkinson's disease OH: orthostatic hypotension; PD: Parkinson's disease; BP: blood pressure; SCOPA-AUT: Scales for Outcomes in Parkinson's disease - Autonomic Dysfunction; COMPASS: Composite Autonomic Symptom Scale; OHQ: Orthostatic Hypotension Questionnaire; OGS: Orthostatic Grading Scale

The clinical assessment of suspected OH should be based on a good history, including the onset and duration, specific characteristics of symptoms on standing, or change of posture (Figure 1) [13,36]. It is also important to remember that the prevalence of OH increases with age [55]. Symptoms can range from dizziness and lightheadedness to visual disturbances (such as blurring or dimming), weakness, fatigue, and cognitive impairment, often referred to as “brain fog” [36,38]. OH is reported among patients with PD. It has also been connected to faster motor and cognitive decline, a higher fall rate, and increased hospitalizations [56]. Clinicians should ascertain whether these symptoms occur exclusively during upright postures and subside when the patient lies down, thus confirming the postural nature of their presentation [38,57]. Discussing potential trigger events is essential, as prolonged standing, warm environments, large meals, alcohol, and certain medications often provoke orthostatic symptoms [41,54,58]. For example, the early morning hours are typically the most symptomatic due to overnight fluid shifts [59]. The physical examination should include meticulous BP and HR measurements in the supine and standing positions, ahead of three minutes of standing, which will allow quantitation of the BP drop and identification of any concomitant supine hypertension [40]. A neurological examination is also paramount in ruling out other conditions, such as ataxia, Parkinsonism, or peripheral neuropathy [60].

An essential point in diagnosing OH is the differential diagnosis between neurogenic and non-neurogenic forms (Table 2). Neurogenic orthostatic hypotension is due to a deficiency of norepinephrine release due to damage to sympathetic nerves, often occurring in synucleinopathies such as PD, multiple system atrophy, and pure autonomic failure (PAF) [38,61]. Evidence has singled out neurogenic orthostatic hypotension as an early manifestation of synucleinopathies, possibly many years before the manifestation of motor symptoms [62,63]. In contrast, non-neurogenic orthostatic hypotension has been attributed to a wide variety of causes, essentially anything from volume depletion, heart problems such as heart failure and arrhythmias, medications, and other medical diseases [64-66]. Recognizing this distinction has implications for choosing effective treatment strategies since therapies for neurogenic orthostatic hypotension typically focus on enhancing norepinephrine signaling or counteracting its deficiency, as noted by Kaufmann [67]. Several clinical clues, outlined in Table 2, can assist clinicians in differentiating between these two forms. Notably, HR response to standing is a key indicator: minimal or absent HR increase in the presence of a significant BP drop strongly suggests neurogenic orthostatic hypotension [39,68]. Neurogenic orthostatic hypotension can be effectively ruled out if the HR increases by at least 0.5 beats per minute for each millimeter of mercury reduction in SBP following three minutes of head-up tilt, making this a reliable marker in clinical assessment [57].

**Table 2:** Key distinguishing features of neurogenic versus non-neurogenic orthostatic hypotension ADH: anti-diuretic hormone (vasopressin); ANS: autonomic nervous system; BP: blood pressure; MSA: multiple system atrophy; NE: norepinephrine; PAF: pure autonomic failure; PD: Parkinson’s disease; RAAS: renin-angiotensin-aldosterone system; SBP: systolic blood pressure; OH: orthostatic hypotension

Feature	Neurogenic OH	Non-neurogenic OH	References
Epidemiology	Prevalence increases with age and is more frequent in those with PD, MSA, or PAF.	Common in older individuals, particularly those with comorbidities.	Velseboer et al. (2011) [32]; Goldstein et al. (2015) [69]; Masaki et al. (1998) [70]
Pathophysiology			
ANS	Central or peripheral autonomic failure; baroreflex failure,	ANS intact; baroreceptor reflex functional	Kaufmann et al. (2017) [71]
Hormonal	Decreased plasma NE, impaired RAAS, and abnormal ADH,	Plasma NE usually rises upon standing, and RAAS and ADH respond appropriately to hypotension.	Goldstein et al. (2015) [69]
Vascular reactivity	Impared vasoconstriction	Preserved vasoconstriction	Kaufmann et al. (2017) [71]
Cardiac autonomic function	Cardiac sympathetic denervation (cardiac scintigraphy)	Normal cardiac sympathetic innervation	Goldstein et al. (2015) [69]
Heart rate response	Blunted or no increase in standing despite the drop in BP (< 0.5 bpm per mmHg SBP drop).	Marked increase when standing as a compensatory mechanism (> 0.5 bpm per mmHg SBP drop).	Norcliffe-Kaufmann et al. (2017) [71]; Smit et al. (1999) [72]
Timing of BP Drop	Usually develops gradually. Often within the first 3 minutes of standing or tilt.	Variable; may be immediate, delayed, or situational.	Kaufmann et al. (2017) [71]
Causes	Damage to sympathetic nerves (Parkinson’s, Multiple System Atrophy, Pure Autonomic Failure).	Dehydration, heart conditions, medications, and anemia.	Freeman et al. (2008) [38]; Robertson et al. (1994) [64]
Other autonomic symptoms	Other autonomic dysfunctions often co-exist with constipation, urinary issues, erectile dysfunction, impaired thermoregulation, etc.	Generally not present, symptoms tend to focus on the single underlying cause (e.g., dehydration).	Ziemssen et al. (2010) [73]; Low et al. (2008) [65]

Diagnostic Tests

Diagnosing OH hinges on identifying a persistent reduction in BP upon transitioning to an upright position [38]. This process encompasses a detailed medical history, a thorough physical examination, and, if necessary, a selection of specialized diagnostic tests [67,74]. Obtaining an appropriate history is key, including investigating the patient's symptom experience: the time of onset, duration, character, and any identifiable triggers or exacerbating factors [36,41]. OH presents with traditional symptoms, including lightheadedness or dizziness, feeling faint, weakness, fatigue, visual blurring, and cognitive difficulties [38].

The active standing test is a fundamental diagnostic tool that accurately measures the HR and BP response in both supine and standing positions [75,76]. OH will be diagnosed if the SBP falls by at least 20 mmHg or the mean DBP drops by at least 10 mmHg within three minutes of standing or head-up tilt. Notably, some patients experience delayed OH where this decrease in BP occurs beyond three minutes, thus necessitating longer monitoring time [40,60]. In a study of 132 newly diagnosed PD patients, 14% exhibited delayed (DOH), while 42% had classical OH (COH). Both DOH and COH were associated with more severe hyposmia compared to patients without OH, suggesting a link between olfactory dysfunction and autonomic impairment. While norepinephrine (NE) levels were reduced in COH, they remained relatively preserved in DOH, indicating less peripheral sympathetic dysfunction. In contrast, vasopressin (ADH) levels were elevated in DOH, possibly reflecting a compensatory mechanism [77].

In addition to the active standing test, various diagnostic tools can help identify the underlying causes of OH and guide effective management strategies [78]. Such multifactorial assessment is often carried out with the use of specialized tools. ABPM recordings provide a continuous record of changes in BP over the 24 hours, outlining overall patterns of changes in BP during the day and night and may point out cases of nocturnal hypertension and OH episodes that would not be picked up by one office visit [79-81]. More importantly, ambulatory blood pressure monitoring (ABPM) can identify symptomatic OH and pressor agent administration times and needs [82].

Abnormal autonomic function testing provides an exact transition of the autonomic nervous system, especially how appropriately the sympathetic and parasympathetic branches respond to certain stimuli. Therefore, as we mentioned before, it is essential to differentiate neurogenic from non-neurogenic causes [83]. This testing also follows standardized protocols or techniques, such as the Valsalva maneuver, HR variability assessments, and the tilt-table test, providing specific insights into autonomic nervous system functioning [84]. Additional autonomic function testing may include sudomotor testing, a widely available and reliable method to determine postganglionic sympathetic cholinergic function, mainly examining sweating integrity. Quantitative sensory testing evaluates small-fiber neuropathy commonly encountered in disorders with autonomic features [78].

Functional near-infrared spectroscopy (fNIRS) combined with convolutional neural networks (CNN) offers a promising approach for evaluating OH in PD. This method effectively analyzes resting-state fNIRS data, achieving over 85% accuracy, which increases to over 90% with the inclusion of correlation matrix inputs [85]. CNN-based fNIRS analysis outperforms traditional machine learning methods, providing a non-invasive and accurate diagnostic tool that reduces patient discomfort and risks linked to conventional tests like the head-up tilt test.

A large cohort study involving 318 PD patients compared the diagnostic accuracy of sit-to-stand versus supine-to-stand BP tests. The study found OH in 35.8% of patients using the supine-to-stand method, significantly outperforming the sit-to-stand test, which had a low sensitivity of 0.39. OH was associated with older age, lower body mass index, longer disease duration, more severe motor and cognitive symptoms, overactive bladder symptoms, functional disabilities, and reduced fluid intake. Notably, three-quarters of OH cases were neurogenic, with many also exhibiting supine hypertension. Continuous BP monitoring further identified OH in 25% of patients missed by clinic-based tests [86].

OH has been increasingly recognized as a key non-motor feature linked to neuronal injury in early-stage PD. Marques et al. demonstrated that serum neurofilament light chain (NfL) effectively distinguishes atypical Parkinsonism disorders (APDs) from PD, with significantly higher NfL levels in APDs, achieving 91% diagnostic accuracy [87]. Park et al. revealed that elevated plasma NfL levels, a neuroaxonal damage biomarker, have been associated with OH in PD, suggesting more extensive neurodegeneration in affected patients [88].

Blood tests can identify anemia, diabetes, or thyroid dysfunction contributing to the problem [64]. Measuring plasma NE levels can help to differentiate the source of nOH, showing postganglionic damage due to decreased norepinephrine versus a preganglionic origin where blood norepinephrine levels are normal or high [62]. Electrocardiography is also utilized to discover occult disturbances in heart rhythm, such as atrial fibrillation, which may contribute to or even mimic symptoms of OH; thus, it plays an essential role in diagnosis and treatment [60,62]. In the tilt-table test, a patient is strapped to a motor-driven table passively tilted upright while monitoring BP and HR under controlled conditions [48]. This controlled approach makes it a valuable tool for identifying delayed OH and orthostatic intolerance in general, helping clinicians distinguish between types of syncope that present with OH (Table 3).

**Table 3:** Diagnostic tests to evaluate orthostatic hypotension. ABPM: ambulatory blood pressure monitoring; ANS: autonomic nervous system; BP: blood pressure; ECG: electrocardiogram; HR: heart rate; nOH: neurogenic orthostatic hypotension; OH: orthostatic hypotension; QST: quantitative sudomotor axon reflex test.

Diagnostic Test	Description	Use in OH	References
Active standing test	Measuring BP and heart rate in supine and standing positions	Essential for diagnosis; confirms a drop in BP upon standing	Shibao et al. (2012) [75]
ABPM	Continuous BP monitoring over 24 hours	Provides a comprehensive picture of BP fluctuations, detects nocturnal hypertension, identifies OH in daily life, and guides treatment decisions.	Kaufmann et al. (2014) [79]
Autonomic function tests	Assess different aspects of ANS function, including heart rate variability, Valsalva response, tilt table test, sudomotor testing, and QST.	It helps distinguish neurogenic from non-nOH and reveals ANS dysfunction. Provides subtype-specific information	Freeman (2014) [83]; Cheshire et al. (2021) [84]; Low et al. (2013) [78]
Blood tests	Evaluate contributing factors (anemia, diabetes, thyroid) and measure norepinephrine	Reveals potential causes of OH; norepinephrine levels can pinpoint the source of nOH.	Robertson et al. (1994) [64]; Goldstein et al. (2007) [62]
ECG	Records the electrical activity of the heart	Detects cardiac arrhythmias that might contribute to or mimic OH	Sarasin et al. (2002) [89]; Cheshire et al. (2021) [84]
Tilt-table test	Gradual tilt to the upright position while monitoring BP and HR under controlled conditions	Evaluates orthostatic intolerance; helps diagnose delayed OH, initial OH, neurally mediated syncope, and postural orthostatic tachycardia syndrome	Thijs et al. (2021) [48]

Grading Systems of Orthostatic Hypotension in Parkinson’s disease

OH is a common non-motor symptom in PD and is estimated to affect a significant proportion of patients [90]. It is a debilitating condition characterized by a drop in BP on standing and may be accompanied by symptoms such as dizziness and lightheadedness, sometimes resulting in syncope [91]. Several grading systems have been developed to improve the understanding and management of OH in PD, broadly categorized into two types: screening tools designed to detect the presence of OH, as previously discussed, and rating scales aimed at quantifying the severity and frequency of associated symptoms [92]. 

Scales for OH-related symptoms give interesting information about their severity as well. The SCOPA-AUT (Scales for Outcomes in Parkinson's disease - Autonomic Dysfunction) and the COMPASS (Composite Autonomic Symptom Scale) are recommended, but with limitations. SCOPA-AUT is a heavily used questionnaire of 25 self-administered items, covering most autonomic dysfunctions: cardiovascular, gastrointestinal, urinary, thermoregulatory, pupillomotor, and sexual [93,94]. OH symptoms are assessed using three items, which provide a good assessment of their presence and frequency [93]. However, despite demonstrating excellent clinometric properties in recent validation studies involving 387 PD patients, its sensitivity for detecting milder orthostatic symptoms is considered limited [92,95]. Also, the COMPASS questionnaire is much more extended, with 73 items covering nine autonomic domains [96,97]. Their nine-item orthostatic symptom subscale correlated with the objective Composite Autonomic Severity Score (CASS) [97,98]. However, the length and complexity of the instrument serve as a barrier to its broad utilization in clinical practice [96].

Beyond the abovementioned instruments recommended for OH assessment, the following scales are frequently used, providing information about the condition. The OH Questionnaire (OHQ) assesses symptom burden and severity using ten items widely validated in different patient populations. It is subdivided into the OH Symptom Assessment and OH Daily Activity Scale [99,100]. It is useful specifically for OH in PD.

The Orthostatic Grading Scale (OGS) consists of five items that measure symptom frequency and severity and the effects of OH on daily life activities [101]. The OGS has the advantage of having a good correlation with the CASS, providing a reliable assessment of autonomic function and orthostatic symptoms [101]. It is also the case that some scales perform single-item screening for OH without rating severity. For example, two relevant questions are part of the assessment of the 30-item non-motor symptom burden in PD in the NMSQuest [102,103]. In this respect, it would represent a beneficial initial screening for identifying potential OH and further and more detailed investigations where necessary [92]. Moreover, the original Unified Parkinson’s Disease Rating Scale (UPDRS) included only the assessment of orthostatic dizziness. Its revised version, the Movement Disorder Society-Unified Parkinson’s Disease Rating Scale (MDS-UPDRS), maintains this approach. While both scales capture a key symptom, they were not validated as stand-alone instruments for evaluating the severity of OH. Therefore, they serve primarily as screening tools rather than comprehensive assessment measures [92,104].

Beyond these scales, there is another scale clinicians commonly use for assessing autonomic dysfunction, including orthostatic symptoms explicitly related to PD: the Scale for Outcome in PD-Autonomic [93]. This tool is predominantly presented as a general assessment of autonomic involvement in PD. It has not been explored for its potential to delineate the severity of OH. Another tool of interest is the Novel Non-Motor Symptoms Scale for PD, which provides a somewhat different perspective on non-motor symptoms and seems to focus on orthostatic symptoms related to syncope and fainting [105]. While this scale has not had much broader application, its presence underlines the expansion of interest in these symptoms, reflecting a significant part of the patient experience [105].

Grading systems for OH in PD will evolve further, providing clinicians with a wide array of assessment tools, including comprehensive scales and concise screening instruments [65,106]. However, further research and validation are needed to enhance their sensitivity and applicability across diverse PD populations. This will aim to standardize and reliably manage OH and eventually enhance patient well-being.

Management

Clinical Versus Nonclinical Significance

Asymptomatic OH patients should be followed up on and treated; a study found that asymptomatic OH was linked to impairments in activities of daily living (ADLs), instrumental IADLs, and ambulatory capacity measures (ACM) comparable to those of symptomatic OH. Regardless of postural lightheadedness, those findings suggest screening for OH in patients with PD [28].

The clinical significance of asymptomatic OH remains uncertain [28]. Orthostatic symptoms may not directly relate to the exact BP measurement or the extent of the BP decrease [107]. Moreover, relying exclusively on patient-reported symptoms when standing to determine whether to treat OH may overlook those who have undetected orthostatic cognitive fluctuations, which can happen without apparent symptoms [46,108]. While not many studies directly compare asymptomatic and symptomatic OH in PD, studies indicate that both groups have similar levels of ambulatory and functional capacity, falls, and healthcare consumption [28,31]. Therefore, failing to address recurrent instances of asymptomatic cerebral hypoperfusion may lead to a gradual decline in cognitive function [109]. Multiple longitudinal studies examining the correlation between OH and cognition in people with PD have discovered that OH is linked to a deterioration in cognitive function [90,110].

OH is frequently observed in PD, with studies reporting a prevalence of 30-50%, though many patients remain asymptomatic [32]. One study of 250 PD patients found measured OH in 30% and subjective orthostatic symptoms in 44%, with the latter linked to older age, dementia, and L-dopa use. Interestingly, measured OH did not correlate with clinical features or specific medications [111]. While lowering both sitting and standing SBP, dopaminergic therapy did not increase OH incidence, likely due to reduced motor tone rather than direct vascular effects [13]. Seasonal variations had no impact on BP measurements [111]. 

Multiple reasons can contribute to the failure to diagnose nOH. One factor is that symptoms of lightheadedness may be absent. In addition, individuals may have more modest symptoms, such as cognitive deceleration [112]. Furthermore, a patient may exhibit no symptoms due to the expansion of the autoregulated range [44]. Several studies have indicated a correlation between OH and higher mortality rates. Two meta-analyses demonstrated an increased risk of all-cause mortality (p < 0.001) in subjects with OH compared with those without this condition [113,114].

Nonpharmacological Interventions

**Figure 2:**
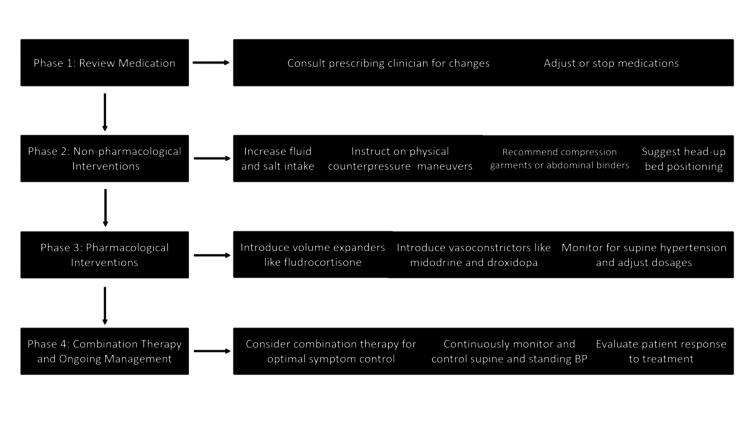
Four-phase treatment strategy of orthostatic hypotension in Parkinson's disease BP: blood pressure

Depending on the severity of OH symptoms, non-pharmacological and pharmacological interventions should be implemented gradually if no reversible cause of OH can be found or if symptoms of OH remain after potentially aggravating variables have been eliminated [115] (Figure 2).

Patients should refrain from alcohol consumption, as alcohol exacerbates venous pooling and can blunt sympathetic vasoconstrictor responses, thereby worsening OH. Additional precautions include avoiding extended standing, heat exposure, large meals high in carbohydrates, and Valsalva-like motions during bowel movements or micturition [116]. Particularly after spending much time lying supine, patients should rise slowly. In this case, they may remain seated before standing up. Daily routine adjustments may be required, such as sitting while peeing or taking a shower in a chair for male patients; if dizziness prevents you from sitting or lying down, try BP-raising techniques by bending forward, crossing your legs, tensely flexing your gluteal and abdominal muscles, or clenching your fists. A training session under continuous BP monitoring may help teach the patient the proper form for these motions and assist them in selecting the most beneficial one [116]. Sleep with the entire body tilted 10 to 20 degrees to promote antidiuretic hormone synthesis throughout the night [117].

One of the most critical non-pharmacological ways to treat OH is to increase the daily intake of salt (6-10 g) and water (up to 2.5 l) [118]. 500 ml of water consumed as a bolus causes a 30 to 90-minute rise in BP [119]. Patients with established heart, renal, or liver failure should take caution while supplementing with salt and water. Additional solutions that have been suggested are predicated on data about distinct elements, such as eating fewer carbohydrates regularly, which may reduce the postprandial hypotension component [120]. Food has been proposed to reduce BP in autonomic failure by insulin secretion and vasodilatation [120,121]. It has been proposed to abstain from alcohol during the day [122,123].

Abdominal binders also alleviate OH by decreasing splanchnic venous pooling [16,124]. In contrast, compression stockings did not show any benefit and could be challenging for the elderly [119,125].

Medications that cause vasodilatation, decrease intravascular volume, or inhibit norepinephrine release or activity at the neurovascular junction aggravate OH and exacerbate symptoms. Often prescribed for benign prostatic hypertrophy, α-blockers, nitrates, phosphodiesterase-5 inhibitors (sildenafil for erectile dysfunction), centrally acting α2‐agonists (clonidine), calcium-channel blockers, beta-blockers, and tricyclic antidepressants are among the common offenders. A personalized risk-benefit analysis may lead to considering a dose change for L-dopa and dopamine agonists, which may also lower BP. On the other hand, there is rarely a correlation between angiotensin-converting enzyme inhibitors and higher BP [55,126-128].

Anemia from chronic diseases is more common in patients with non-OH [129]. Because anemia lowers blood viscosity and oxygen-carrying capacity, it exacerbates OH. Notably, hemoglobin scavenges nitric oxide, a potent vasodilator [130]. It is likely, though still unproven, that nitric oxide-mediated processes exacerbate vasodilation in anemic patients with nOH, just as they do in other illnesses [131]. Anemia needs to be appropriately diagnosed and treated. Recombinant erythropoietin therapy has been shown in small case series to reduce BP while standing up [132]. OH can also be brought on or made worse by a vitamin B12 deficiency (<250 pg/mL with high methylmalonic acid levels), which must be treated [133].

Acupuncture at the stellate ganglion appears to be an effective adjunctive therapy for OH in PD, offering benefits beyond standard pharmacological treatment. In a randomized study of 68 PD patients with OH, those receiving a combination of acupuncture and midodrine hydrochloride showed more significant improvements in orthostatic SBP and DBP, alongside substantial reductions in Orthostatic Hypotension Questionnaire scores, Traditional Chinese Medicine syndrome scores, and UPDRS scores compared to those receiving medication alone. Additionally, serum NE levels increased significantly in the combination group, suggesting enhanced sympathetic activation as a potential mechanism. The total effective rate was notably higher in the combination group (87.1%) than in the medication-only group (63.6%), supporting stellate ganglion acupuncture's potential role in improving hemodynamic stability and clinical symptoms in PD-related OH [134].

The effects of deep brain stimulation (DBS) on the cardiovascular system of patients with resistant neurological disorders are still unknown [135]. In a study of 20 PD patients, cardiovascular reflex tests showed no significant differences in BP control before and six months after subthalamic nucleus (STN)-DBS in the medication-off state. However, after levodopa intake, the drop in BP during the head-up tilt test was more pronounced both pre- and post-DBS. Notably, levodopa-induced OH was observed in 25% of patients before DBS, decreasing to 5% post-DBS [136]. These findings suggest that while STN-DBS does not directly impact autonomic cardiovascular control, it may reduce the frequency of levodopa-induced OH, improving BP stability in PD patients.

Pharmacological Interventions

Pharmacological management of OH in PD seeks to ameliorate symptomatic burden and enhance functional capacity; however, therapeutic benefits must be carefully weighed against potential adverse effects. Fludrocortisone, a mineralocorticoid agonist, carries risks of exacerbating supine hypertension and may induce hypokalemia or peripheral edema [65]. Pyridostigmine, an acetylcholinesterase inhibitor, offers the advantage of improving orthostatic tolerance without aggravating supine hypertension, though its use may be limited by cholinergic adverse effects such as diarrhea and nausea [137,65]. The α1-adrenergic agonist midodrine effectively augments peripheral vascular resistance to elevate BP, but its utility may be constrained by bothersome adrenergic effects, including piloerection, paresthesias, pruritus, and hypertension [138]. The norepinephrine precursor droxidopa (L-DOPS) demonstrates favorable tolerability with predominantly mild adverse effects, though vigilant monitoring for supine hypertension remains warranted. Optimal therapeutic selection requires individualized risk-benefit assessment tailored to patient-specific factors and comorbidities (Table 4) [139].

**Table 4:** Management of orthostatic hypotension in Parkinson’s disease BP: blood pressure; DBP: diastolic blood pressure; FC: fludrocortisone; MBP: mean blood pressure; OH: orthostatic hypotension; PD: Parkinson’s disease; SBP: systolic blood pressure; TID: three times a day; NA: not applicable.

Drug	Recommendations	Results	n	Reference
Fludrocortisone	14 days 3 × 60 mg/day pyridostigmine bromide or 1 × 0.2 mg/day fludrocortisone	Significant improvement in both diastolic BP drop on the orthostatic challenge (−37%) and MBP standing (+15%) by 0.2 mg/day FC in PD	13	Schreglmann et al. (2017) [149]
Pyridostigmine	A single 60-mg dose of pyridostigmine bromide, alone or in combination with a subthreshold (2.5 mg) or suprathreshold (5 mg) dose of midodrine hydrochloride	Pyridostigmine significantly improves standing BP in patients with OH without worsening supine hypertension. The greatest effect is on diastolic BP, suggesting that the improvement is due to increased total peripheral resistance.	58	Singer et al. (2006) [137]
Acarbose	100 mg of acarbose taken 20 minutes before meals	Acarbose reduced the postprandial fall in SBP and DBP by 17 mm Hg and nine mm Hg, respectively, compared with the placebo.	13	Shibao et al. (2007) [150]
Midodrine	2.5, 10, 20 mg of midodrine	Midodrine may improve standing SBP and global symptoms but has no significant benefit on supine to standing SBP and MBP and causes a higher incidence of adverse events.	325	Parsaik et al. (2013) [151]
Atomoxetine	The patients received either atomoxetine (18 mg daily) or midodrine (5 mg twice daily).	Atomoxetine improved the standing SBP and DBP drop after one month of treatment, which was comparable to that achieved with midodrine therapy.	50	Byun et al. (2019) [152]
Droxidopa	The mean droxidopa dose during the double-blind administration period was 429 ± 163 mg, with 38% of patients (92/244) taking the maximum dosage of 600 mg 3 times daily.	Treatment with droxidopa significantly increased upright SBP compared with placebo (11.5 ± 20.5 vs. 4.8 ± 21.0 mmHg; P < 0.001). A significant increase in upright DBP was also noted for droxidopa versus placebo (8.0 ± 15.5 vs. 1.8 ± 17.3 mmHg; P < 0.001).	460	Biaggioni et al. (2017) [153]
Domperidone	10, 20, and 50 mg TID	Although not statistically significant, several studies have identified a trend toward symptom improvement when apomorphine is used as adjunctive therapy with domperidone	NA	Bacchi et al. (2017) [154]
Devices				
Compression bandage of legs and abdomen	The patient received a 10-minute leg bandage (compression pressure of 40 to 60 mm Hg), followed by an additional 10-minute abdominal bandage (20 to 30 mm Hg).	Lower limb compression bandages effectively prevent orthostatic systolic BP decrease and reduce symptoms in elderly patients with progressive orthostatic hypotension.	21	Podoleanu et al. (2006) [155]

Given the uncertainty surrounding its long-term safety, the pharmacological treatment of nOH is complicated and should be cautiously administered [140]. It should be considered for pharmacological treatment, which consists of pressor medications and intravascular volume expansion [13,141]. Clinical guidelines from the European Federation of Neurological Societies (EFNS) and expert consensus aligned with the American Academy of Neurology (AAN) recommend a stepwise approach for managing OH in Parkinson’s disease. Initial strategies include fluid and salt intake, physical counter-maneuvers, and compression therapy, followed by pharmacologic agents such as midodrine, fludrocortisone, and droxidopa when needed [142,143]. Fludrocortisone initially causes the extracellular fluid volume to expand, raising BP [144]. Compared to mineralocorticoid agonists, pressor medications, such as the synthetic norepinephrine precursor droxidopa and midodrine, have a shorter half-life and are safer [145]. On the other hand, when peripheral sympathetic neurons are functioning, like in the case of individuals with preganglionic or premotor sympathetic lesions, such as multiple system atrophy, noradrenaline-reuptake inhibitors, like atomoxetine, have a pressor effect [146]. When taken alone, the acetylcholinesterase inhibitor pyridostigmine has only mild vasopressor effects [146,147]. The α-glucosidase inhibitors acarbose and caffeine can be used to treat postprandial hypotension [13,141,148].

Fludrocortisone is a typical synthetic mineralocorticoid used to treat hypotension. It increases water retention and sodium reabsorption rates. Research has demonstrated that in individuals with nOH brought on by diabetic neuropathy or PD, fludrocortisone raises SBP, alleviates symptoms, and reduces orthostatic tachycardia [149,156]. Headaches, peripheral edema, and supine hypertension are the most frequent side effects. Fludrocortisone is contraindicated in treating patients with hypertension, hyperalbuminemia, and systemic fungal infections [156].

Pyridostigmine, an acetylcholinesterase inhibitor, is used off-label to treat nOH. Its effect is thought to stem from enhanced sympathetic ganglionic transmission (due to increased cholinergic neurotransmission) rather than direct vasoconstriction [137]. Under orthostatic stress, this elevated cholinergic tone may increase norepinephrine release [137,157]. In a comparative experiment, fludrocortisone appeared more successful than pyridostigmine bromide in increasing peripheral systolic supine BP and mean arterial BP [149]. Pyridostigmine also caused supine SBP, but not as much as midodrine did. A combination trial of midodrine and pyridostigmine improved the DBP and SBP, making it the most effective treatment for hypotension [137,158]. Researchers observed adverse effects that included headaches, vertigo, gastrointestinal distress, tremors in the limbs, and possibly even sadness, apathy, and insomnia [158]. Similarly, pyridostigmine alone was not as effective as pyridostigmine in combination with other drugs, such as atomoxetine and propranolol or bisoprolol [159,160].

Acarbose functions as an inhibitor of α-glucosidase. Due to its ability to slow down the small intestine's enzymatic breakdown of carbs, it is mainly utilized in treating type 2 diabetes. Due to its efficacy in reducing the dip in BP that follows meals, acarbose could also be used to manage PPH in individuals with chronic autonomic failure. Lower plasma glucose levels brought on by acarbose treatment may decrease plasma insulin levels. Lowering the plasma levels of insulin, a known vasodilator, lowers PPH [150]. Occasional cases of reversible elevations in liver transaminase levels have been reported [161].

The US Food and Drug Administration (FDA) has approved midodrine as a medication for the treatment of OH. Midodrine is an α1 agonist that causes increased peripheral vascular resistance and vasoconstriction, leading to increased BP [162]. According to reports, midodrine raises standing SBP, lessens sensations of dizziness, and enhances investigators' and patients' overall symptom alleviation scores [163]. Furthermore, it has been observed that midodrine reduces intradialytic hypotension symptoms [162]. Pilomotor responses, urine retention, and supine hypertension are the most frequent side effects reported by midodrine users [163]. Patients with spinal cord injuries who were previously hypotensive and were given 10 mg of midodrine experienced a considerable increase in BP and a decrease in hypotensive episodes [164].

The FDA has approved atomoxetine, a selective norepinephrine transporter blocker, to treat attention deficit hyperactivity disorder (ADHD) [60]. It has a well-established track record of long-term effectiveness and safety in treating ADHD patients [165]. Orthostatic BP drop and OH-related symptoms in patients with non-OH have been successfully treated with atomoxetine at a daily dose of 18 mg [159]. Due to its potential to improve norepinephrine availability in the synaptic cleft, it has been used off-label to control nOH [60].

A prodrug of norepinephrine is droxidopa [166]. The administration of droxidopa, a synthetic amino acid that the enzyme L-aromatic amino acid decarboxylase converts to norepinephrine, raises standing BP, reduces OH symptoms, and enhances a patient's capacity to adjust posture in individuals with non-OH conditions caused by degenerative autonomic disorders such as PD [139]. Droxidopa has only incidental effects. Headache, lightheadedness, nausea, and infrequently neuroleptic malignant syndrome are typical adverse effects of this medication [166].

Current Clinical Trials

It is important to emphasize that the initial line of treatment for OH is always nonpharmacologic. The effect of drinking water on BP in patients with nOH was initially investigated in a study with 28 patients with multiple system atrophy (MSA), 19 patients with PAF, and 19 healthy individuals as controls. This study was conducted in an open-label manner. Following 30 minutes of consuming 500 ml of tap water, the seated SBP and DBP increased by an average of 33 ± 5/16 ± 3 mmHg in patients diagnosed with MSA and by 37 ± 7/14 ± 3 mmHg in patients with PAF (p < 0.0001 compared to the initial measurements in both illnesses). The rise in SBP lasted for more than 60 minutes [167,168].

In another clinical trial investigating nonpharmacologic interventions, a total of 15 patients diagnosed with PD and OH were randomly assigned to either use a compressive abdominal binder (20 mmHg of abdominal pressure) or a sham abdominal binder (3 mmHg of abdominal pressure) during a head-up tilt procedure [16]. The main objective was to measure the average BP following three minutes of head-up tilt. The compression abdominal binder resulted in a rise of 7.7 mmHg, while the sham abdominal binder caused a decrease of -2.7 mmHg. Subsequently, there was a four-week period wherein all patients used the compression abdominal binder. This led to a reduction of orthostatic symptoms, as indicated by a drop of 2.2 points on the OH Questionnaire (OHQ) compared to the initial measurement (p=0.003) [16].

An open-label clinical study was conducted recently in which 87 patients with OH were randomly assigned to one of three therapy groups: midodrine 2.5 mg only, pyridostigmine 30 mg solo, or a combination of midodrine 2.5 mg and pyridostigmine 30 mg. The primary outcome measure was the change in BP (ΔBP) within three minutes of standing, assessed three months after treatment. The secondary objectives included assessing the ΔBP after three minutes of standing after one month and the OH Questionnaire (OHQ). The authors observed that the SBP and DBP changes were less severe following therapy, independent of the specific medication used. This experiment had a significant bias in multiple aspects: it lacked blinding; despite the authors' claim of exclusively including patients with nOH, no confirmatory tests were conducted, allowing for the potential enrolment of patients with OH from other causes [157].

The study was a randomized, single-masked, crossover trial involving 65 individuals with nOH. The trial compared the effects of a single atomoxetine dosage (18 mg) with midodrine (5-10 mg). The primary measure of interest was the SBP while standing, one minute, and one hour after administering the medication. The secondary endpoints assessed in the study were posttreatment seated SBP and DBP, standing DBP, and HR, as well as scores for the OH questionnaire (OHQ) and OHQ item 1. Atomoxetine caused a substantial increase in standing SBP by 20 mmHg and DBP by 11 mmHg compared to the placebo. This difference was statistically significant, with a p-value of less than 0.001 in both cases. In addition, midodrine caused a considerable increase in standing SBP of 12 mmHg and standing DBP of 7 mmHg compared to the placebo (p < 0.001 for both). Atomoxetine demonstrated a more significant improvement in standing SBP than midodrine, with a mean difference of 7.5 mmHg (95% CI, 0.6 to 14.5; p = 0.03) [169].

Droxidopa was authorized for the treatment of nOH based on three randomized clinical trials that showed improvement in OH symptoms [79,170,171]. Supine hypertension, which refers to high BP while lying down, is the most frequent adverse effect of droxidopa. However, the occurrence of this side effect is relatively low, with a rate of ≤7.9% compared to ≤4.6% for a placebo [172]. It is worth noting that droxidopa is believed to be safer than midodrine in terms of causing supine hypertension. A recent meta-analysis found that midodrine, but not droxidopa, significantly increases the risk of supine hypertension [173].

Pathophysiology

Neuropathology

**Figure 3:**
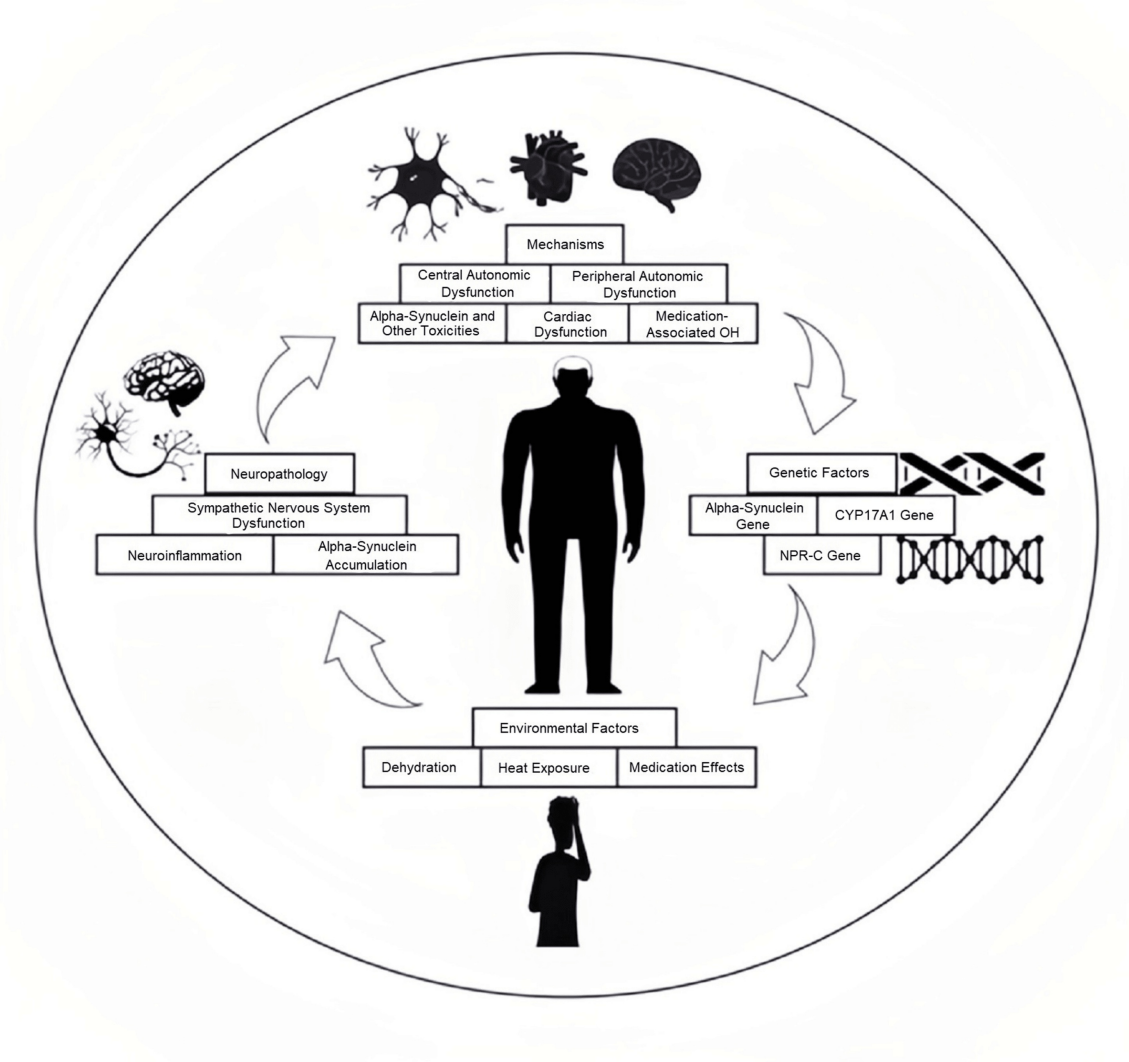
Complex factors underpinning orthostatic hypotension in Parkinson's disease OH: orthostatic hypotension Original figure created by the authors.

The sympathetic nervous system is essential for preserving stable BP (Figure 3). A crucial component of this system is the release of norepinephrine, a neurotransmitter that instructs blood vessels to contract, by postganglionic efferent sympathetic neurons. The degeneration of these particular neurons results in inadequate release of norepinephrine in diseases such as PD. Consequently, this impedes the essential vasoconstriction upon standing, leading to a decrease in BP called OH [174].

Alpha-synuclein accumulation in Lewy bodies, the pathological hallmark of PD, disrupts autonomic function, particularly in brain regions governing sympathetic activity. This disruption leads to OH, a common and debilitating symptom characterized by a significant drop in BP upon standing. Additionally, alpha-synuclein accumulation triggers chronic inflammation in the brain and can spread to other regions, contributing to the progressive nature of PD and its diverse clinical manifestations [175].

Neuroinflammation, a hallmark of PD, particularly in the brainstem, is implicated in the development of OH. This complication arises from the detrimental effects of activated microglia and pro-inflammatory cytokines on the nucleus tractus solitarius, a critical BP regulatory center. Further investigations are warranted to elucidate the intricate relationship between neuroinflammation and OH in PD, paving the way for novel therapeutic interventions aimed at preventing or mitigating this debilitating condition [176].

Genetic Factors

Several genes are associated with an elevated risk of developing OH in PD. These genes are implicated in various aspects of BP regulation, including the production of norepinephrine, a crucial neurotransmitter responsible for maintaining vascular tone [62]. α-synuclein is a protein known to be associated with PD, and it is also found in Lewy bodies, which are abnormal protein aggregates characteristic of the disease. Goldstein et al. found that individuals with PD and OH had cardiac sympathetic denervation and a loss of functional sympathetic nerves [177]. This suggests that α-synuclein may play a role in developing OH in these patients, as it is involved in the loss of sympathetic nerves. While the α-synuclein gene has been the focus of much research regarding its potential role in OH, other genes may also play a role. Interestingly, patients with GBA gene variants (GBA-PD) to 313 idiopathic PD revealed a more significant drop in SBP during postural changes in the GBA-PD group. In contrast, the groups' DBP and HR remained similar [178].

The CYP17A1 gene encodes the enzyme 17α-hydroxylase, which synthesizes cortisol [179]. Cortisol is a hormone that helps regulate BP, and low cortisol levels can contribute to OH. NPR-C encodes the natriuretic peptide receptor C, which regulates BP by promoting vasodilation and renal sodium and water excretion [177]. Mutations in this gene have been associated with OH.

In a cross-sectional study of 304 PD patients, specific single-nucleotide polymorphisms (SNPs) were found to influence the risk of nOH, which was present in 11.5% of the cohort. SNPs in genes such as SCN3A, MIR4696, LZTS3/DDRGK1, FAM47E-STBD1, TMEM175, SH3GL2, CCDC62, and SNCA were associated with a higher risk of nOH, while variants in SIPA1L2, ITGA8, NDUFAF2, and IP6K2 were linked to a lower risk. These genetic variants were found to affect the expression of genes within key autonomic nervous system structures and influence metabolic and signaling pathways, notably IP3/Ca²⁺ signaling, the PKA-CREB pathway, and fatty acid metabolism [180].

Environmental Factors

Several environmental factors can exacerbate OH in individuals with PD. For instance, dehydration lowers blood volume, making it more difficult for the heart to pump blood at the proper pressure. Heat causes vasodilation, which widens blood vessels and lowers BP. Sweating also causes the body to lose more fluids, intensifying the consequences of heat. Several drugs, including antidepressants, diuretics, and vasodilators, can disrupt the body's normal BP regulation, raising the possibility of OH [181].

Additionally, prolonged standing allows blood to pool in the lower extremities, reducing venous return and BP. Sudden changes in body position, such as standing up quickly, can overwhelm the body's compensatory mechanisms, leading to a transient drop in BP [182]. Critical preventative measures for this illness include drinking enough water, avoiding heated places, and talking to your doctor about changing your prescription [183].

Mechanisms

OH, in PD, is a complex phenomenon with multiple contributing mechanisms. These mechanisms can be broadly categorized into five main areas.

Central Autonomic Dysfunction

Central autonomic dysfunction (CAD), a hallmark of neurodegenerative diseases, plays a critical role in OH by impairing the baroreflex, leading to inadequate BP adjustments upon standing. This dysfunction also manifests as supine hypertension and loss of diurnal BP variation, highlighting the complex autonomic control impairments in these conditions. Understanding the mechanisms of central autonomic dysfunction in OH is crucial for developing effective treatment strategies, including addressing baroreflex impairment, managing associated symptoms like supine hypertension, potentially exploring interventions that target the underlying neurodegenerative processes, and implementing lifestyle modifications to minimize orthostatic stress [184].

The underlying mechanisms of CAD-induced OH in PD are complex and multifactorial. Degeneration of noradrenergic neurons in the brainstem, particularly within the locus coeruleus (LC), plays a pivotal role. This degeneration disrupts the sympathetic nervous system's ability to effectively increase HR and peripheral vasoconstriction upon standing, leading to inadequate BP compensation [185]. However, Palermo et al. found no direct link between LC integrity and OH in PD. While PD patients showed reduced LC signal intensity compared to healthy controls, no significant differences existed between those with and without OH. Moreover, the extent of LC damage did not correlate with orthostatic BP drops or the severity of autonomic symptoms, suggesting that additional mechanisms beyond LC degeneration may contribute to OH in PD [186].

nOH leads to significant orthostatic reductions in cerebral blood flow velocity (CBFv), even in the absence of overt symptoms, potentially contributing to poor neurological outcomes due to subclinical cerebral hypoperfusion. In this context, individuals with nOH experience more significant CBFv declines than healthy controls (Hedge g = -0.64, p < 0.001). However, CBFv reductions did not significantly differ between nOH patients and disease-matched controls without nOH. Importantly, symptomatic nOH patients exhibited more significant CBFv drops than asymptomatic patients (Hedge g = 0.84, p = 0.009), suggesting that symptom severity may reflect more significant cerebral hypoperfusion [187]. Moreover, in PD patients with OH, near-infrared spectroscopy revealed more substantial and prolonged reductions in oxygenated hemoglobin in the left hemisphere during the head-up tilt test and delayed peak time during the squat-to-stand test, despite no significant differences in transcranial Doppler-measured mean flow velocity [188]. However, considerable variability exists in the literature regarding fNIRS findings, with 58% of studies reporting a positive correlation between brain oxygenation changes and BP variations, 3% showing a negative correlation, and 39% finding no correlation [189].

Peripheral Autonomic Dysfunction

Peripheral autonomic dysfunction, characterized by impaired sympathetic nerve activity and reduced vasoconstriction, plays a significant role in the development of OH. This dysfunction, frequently associated with conditions such as diabetes mellitus and PD, manifests as inadequate adjustments in vascular tone upon postural changes, leading to a substantial drop in BP and associated symptoms like dizziness, lightheadedness, and fainting [190-191]. Beyond its role in OH, peripheral autonomic dysfunction also contributes to supine hypertension. This seemingly paradoxical finding underscores the complex interplay between central and peripheral mechanisms in maintaining BP homeostasis. Understanding the mechanisms of peripheral autonomic dysfunction is paramount for developing effective treatment strategies that target both autonomic dysfunction’s central and peripheral components. Such strategies promise to improve BP control and alleviate the debilitating symptoms of OH in affected individuals [190].

Alpha-Synuclein and Other Toxicities

Alpha-synuclein pathology, a hallmark of PD, extends beyond the CNS, affecting the peripheral autonomic nervous system and potentially contributing to OH, a common and debilitating non-motor symptom in PD. The aggregation of alpha-synuclein in sympathetic and parasympathetic nerves may impair norepinephrine release and baroreceptor reflex function, leading to an inability to maintain adequate BP upon standing. Additionally, alpha-synuclein pathology in the enteric nervous system could contribute to OH through impaired fluid and electrolyte absorption. Understanding the link between alpha-synuclein and OH could pave the way for novel therapeutic strategies to prevent or manage this debilitating symptom in PD patients [191].

In PD, mitochondrial dysfunction can lead to impaired autonomic nervous system function, resulting in an inability to adjust BP and HR effectively upon standing, which causes OH. Additionally, mitochondrial dysfunction can increase oxidative stress and damage neurons in the autonomic nervous system, further exacerbating OH symptoms [181].

Cardiac Dysfunction

Cardiac dysfunction stands as a critical factor in the development of hypotension upon standing, a condition marked by a significant drop in BP upon transitioning from a lying to a standing position. This phenomenon arises from the heart's inability to adequately pump blood to meet the body's increased demand upon standing, leading to decreased blood flow to the brain. This insufficient perfusion manifests as dizziness, lightheadedness, and even syncope [184]. Cardiac dysfunction plays a pivotal role in the pathogenesis of OH. Impaired myocardial contractility and reduced stroke volume, hallmarks of heart failure, directly contribute to an inadequate BP response upon standing. This diminishes the ability to maintain adequate tissue perfusion during orthostasis, leading to the characteristic symptoms of OH [193]. A review of 54 studies involving 3,114 Parkinson’s disease patients found that ¹²³I-MIBG scintigraphy has high sensitivity (0.81-0.83) and specificity (0.80-0.86) for detecting cardiac sympathetic denervation [194].

Medication-Associated Hypotension in Parkinson’s disease

Several medications used to treat PD can contribute to OH. Dopaminergic medications like levodopa can increase dopamine levels in the brain, but can cause vasodilation and OH. Also, some anticholinergic drugs, such as benztropine and trihexyphenidyl, may block acetylcholine, affecting BP regulation and potentially leading to OH. Other medications that can also contribute to OH in PD are antidepressants, antipsychotics, and diuretics. The mechanism by which these medications cause OH varies. Dopaminergic drugs can directly cause vasodilation, while anticholinergics can interfere with the autonomic nervous system's ability to regulate BP. Other medications may indirectly affect BP regulation [195].

While hydrochlorothiazide's vasodilatory effect contributes to its antihypertensive action, it can also lead to OH, a condition characterized by a sudden drop in BP upon standing. This hypotensive response can manifest as dizziness, lightheadedness, and even fainting, posing a significant risk for falls and injuries, especially in the elderly population [196].

Expert recommendations and future studies

Experts recommend careful titration and monitoring of PD medications to reduce the risk of OH. This approach includes identifying drugs that may exacerbate OH, adjusting dosages, or substituting them with alternatives that carry a lower risk. Continuous BP monitoring is essential to evaluate these adjustments and detect potential OH episodes.

The severity and response to treatment for OH vary widely among PD patients, highlighting the need for personalized treatment plans. These plans should consider medication regimens, comorbidities, and lifestyle habits. Developing effective customized treatment plans remains challenging, requiring further research to identify reliable predictors of OH risk and response to different interventions.

Patient education regarding OH is crucial to empower individuals with PD to recognize and manage their symptoms effectively. This education should include information about the causes and symptoms of OH, strategies for preventing OH episodes, and appropriate actions to take if OH occurs. Educational materials and resources should be tailored to the individual's needs and cognitive abilities to ensure practical understanding and implementation.

Cardiovascular autonomic dysfunction has emerged as a potential differentiating factor between tremor-dominant PD (TDPD) and essential tremor (ET), conditions that often present overlapping motor symptoms, complicating diagnosis. Recent studies have demonstrated that heart rate variability (HRV) analysis and OH assessment can aid in distinguishing these disorders. In particular, TDPD patients exhibit more frequent OH and a more significant SBP drop during tilt tests compared to ET patients and healthy controls. Additionally, HRV parameters, such as reduced low-frequency power and decreased variability in RR intervals, are commonly impaired in TDPD but remain unaffected in ET. Diagnostic measures like SBP drop and specific HRV metrics have shown strong discriminative power, with area under the curve (AUC) values exceeding 0.8 [197].

Future research on OH in PD should focus on developing and evaluating interventions to improve the quality of life for affected individuals. Potential assessment areas include non-pharmacological interventions like physical therapy, compression garments, dietary modifications, and novel pharmacological agents. Physical therapy programs can improve balance, gait, and muscle strength, while compression garments can improve blood flow and reduce OH symptoms. Dietary changes, such as increasing fluid intake and salt consumption, can also impact OH symptoms and overall well-being. Novel pharmacological agents target specific mechanisms underlying OH in PD, such as agents that improve autonomic nervous system function or enhance vascular tone.

An investigation of combining pharmacological and non-pharmacological interventions to address multiple underlying causes of OH in PD could provide more comprehensive and effective management strategies.

## Conclusions

Orthostatic hypotension is a common, underdiagnosed, and often debilitating non-motor symptom in Parkinson’s disease, resulting from complex interactions involving central and peripheral autonomic dysfunction, alpha-synuclein accumulation, baroreflex failure, and medication effects. This review has outlined the distinct mechanisms and clinical presentations of both neurogenic and classical orthostatic hypotension, highlighting the variability in prevalence and symptom severity among affected individuals. Given its potential to precede motor symptoms and contribute to increased morbidity, early recognition remains critical. Accurate diagnosis relies on careful clinical history-taking, postural blood pressure measurements, and specialized autonomic testing. The distinction between neurogenic and non-neurogenic forms is essential for determining appropriate therapeutic strategies. Initial management should prioritize non-pharmacological approaches such as fluid and salt supplementation, head-of-bed elevation, compression therapy, and physical counter-maneuvers. Pharmacologic agents, including midodrine, droxidopa, and fludrocortisone, are reserved for cases unresponsive to conservative measures. The review also presents data on the utility of grading scales, the implications of asymptomatic hypotension, and the potential of emerging treatments such as atomoxetine and acupuncture. Clinicians should maintain a high index of suspicion, routinely assess for orthostatic changes, and apply individualized management plans to minimize complications and improve patients' autonomy, safety, and quality of life.
